# Seccotine in Surgery

**Published:** 1900-06-23

**Authors:** 


### SECCOTINE IN SURGERY.

A very practical "tip" is given by Dr. James Macaskie[1] in regard to the extraction of foreign bodies from the ear. He was called to see a schoolboy who had pushed into the meatus a piece of indiarubber which had previously been attached to a lead pencil. The rubber had been driven well in, and as it was an almost exact mould of the passage, and presented a perfectly flat surface, it was impossible to catch hold of it, and syringing did not seem likely to improve matters. Dr. Macaskie therefore cleaned away all the wax with ether and then teased out the end of a small piece of twine, and giving this a good coating of seccotine pushed it tightly against the indiarubber, and packed it closely all round with cotton wool. After this had been left in place for 24 hours there was firm cohesion, and not the slightest difficulty was experienced in withdrawing everything *en masse*. In form this expedient is, we believe, not original. The difficulty has been to obtain a material which would adhere to all sorts of things. For this, and similar purposes, seccotine will be found very useful; but Dr. Macaskie's forethought in cleaning with ether no doubt conduced much to his success.

[1] Lancet, June 2.

